# Supplementary material for: Communication between distinct subunit interfaces of the cohesin complex promotes its topological entrapment of DNA
Source: eLife. 2019 Jun 4;8:e46347. doi: 10.7554/eLife.46347 (PMC6579514; doi:10.7554/eLife.46347)
Supplement: Supplementary file 1. [file elife-46347-supp1.docx]

**Supplementary file 1. Yeast strains.**

VG3620-4C *Mat****a*** *TIR1-CgTRP1 LacO-NAT::lys4 GFPLacI-HIS3:his3-11,15 leu2-3,112 ura3-52 bar1*

VG3630-7A *Mat****a*** *G418:SCC2-3V5-AID2 TIR1-CgTRP1 LacO-NAT::lys4 GFPLacI-HIS3:his3-11,15 ura3-52 leu2-3,112 bar1*

VG3808-1A *Mat****a*** *G418:SCC3-3V5-AID2 TIR1-CgTRP1 LacO-NAT::lys4 GFPLacI-HIS3:his3-11,15 ura3-52 leu2-3,112 bar1*

VG3940-2D *Mat****a*** *SMC3-MCD1::∆mcd1 smc3∆::HPH LacO-NAT::lys4 GFPLacI-HIS3:his3-11,15*

*leu2-3,112 ura3-52 bar1*

VG3945-1A *Mat****a*** *G418:SCC2-3V5-AID2* *SMC3-MCD1::∆mcd1 smc3∆::HPH LacO-NAT::lys4 GFPLacI-HIS3:his3-11,15 leu2-3,112 ura3-52 bar1*

VG3946-7B *Mat****a*** *G418:SCC3-3V5-AID2* *SMC3-MCD1::∆mcd1 smc3∆::HPH LacO-NAT::lys4 GFPLacI-HIS3:his3-11,15 leu2-3,112 ura3-52 bar1*

VG3954-10C *Mat****a*** *G418:PDS5-3V5-AID2 TIR1-CgTRP1 LacO-NAT::lys4 GFPLacI-HIS3:his3-11,15 leu2-3,112 ura3-52 bar1*

VG3955-4D *Mat****a*** *G418:PDS5-3V5-AID2* *SMC3-MCD1::∆mcd1 smc3∆::HPH LacO-NAT::lys4 GFPLacI-HIS3:his3-11,15 leu2-3,112 ura3-52 bar1*

VG3930-5C *Mat****a*** *SMC3-K112R,K113R-MCD1::∆mcd1 smc3∆::HPH LacO-NAT::lys4 leu2-3,112*

*GFPLacI-HIS3:his3-11,15 ura3-52 bar1*

VG3651-3D *Mat****a*** *SMC3-3V5-AID^608^ TIR1-CgTRP1 LacO-NAT::lys4 GFPLacI-HIS3:his3-11,15*

*leu2-3,112 ura3-52 bar1*

BRY474 *Mat****a*** *SMC3-LEU2:leu2-3,112 SMC3-3V5-AID^608^ TIR1-CgTRP1 LacO-NAT::lys4 GFPLacI-HIS3:his3-11,15 ura3-52 bar1*

BRY492 *Mat****a*** *smc3-L1029R-LEU2:leu2-3,112 SMC3-3V5-AID^608^ TIR1-CgTRP1 LacO-NAT::lys4 GFPLacI-HIS3:his3-11,15 ura3-52 bar1*

VG3905-7A *Mat****a*** *smc3-I1026R-LEU2:leu2-3,112 SMC3-3V5-AID^608^ TIR1-CgTRP1 LacO-NAT::lys4 GFPLacI-HIS3:his3-11,15 ura3-52 bar1*

VG3943-1C *Mat****a*** *SMC3-6HA^F864^-LEU2:leu2-3,112 SMC3-3V5-AID^608^ TIR1-CgTRP1*

*LacO-NAT::lys4 GFPLacI-HIS3:his3-11,15 ura3-52 bar1*

VG3944-3D *Mat****a*** *smc3-6HA^F864^-I1029R-LEU2:leu2-3,112 SMC3-3V5-AID^608^ TIR1-CgTRP1*

*LacO-NAT::lys4 GFPLacI-HIS3:his3-11,15 ura3-52 bar1*

VG3902-3A *Mat****a*** *G418:MCD1-AID TIR1-CgTRP1 LacO-NAT::lys4 GFPLacI-HIS3:his3-11,15*

*leu2-3,112 ura3-52 bar1*

VG3914-1C *Mat****a*** *MCD1-LEU2:leu2-3,112 G418:MCD1-AID TIR1-CgTRP1 LacO-NAT::lys4*

*GFPLacI-HIS3:his3-11,15 leu2-3,112 ura3-52 bar1*

VG3916-5B *Mat****a*** *mcd1-L75K-LEU2:leu2-3,112 G418:MCD1-AID TIR1-CgTRP1 LacO-NAT::lys4*

*GFPLacI-HIS3:his3-11,15 leu2-3,112 ura3-52 bar1*

VG3918-9D *Mat****a*** *mcd1-L89K-LEU2:leu2-3,112 G418:MCD1-AID TIR1-CgTRP1 LacO-NAT::lys4*

*GFPLacI-HIS3:his3-11,15 leu2-3,112 ura3-52 bar1*

VG3694-7C *Mat****a*** *SMC3MCD1-URA3:ura3-52 SMC3-3V5-AID^608^ TIR1-CgTRP1 LacO-NAT::lys4 GFPLacI-HIS3:his3-11,15 leu2-3,112 bar1*

VG3908-17B *Mat****a*** *smc3-I1026R-MCD1-URA3:ura3-52 SMC3-3V5-AID^608^ TIR1-CgTRP1*

*LacO-NAT::lys4 GFPLacI-HIS3:his3-11,15 leu2-3,112 bar1*

VG3872-3B *Mat****a*** *smc3-L1029R-MCD1-URA3:ura3-52 SMC3-3V5-AID^608^ TIR1-CgTRP1*

*LacO-NAT::lys4 GFPLacI-HIS3:his3-11,15 leu2-3,112 bar1*

VG3937-2C *Mat****a*** *SMC3MCD1-LEU2:leu2-3,112 G418:MCD1-AID TIR1-CgTRP1 LacO-NAT::lys4 GFPLacI-HIS3:his3-11,15 ura3-52 bar1*

VG3938-3A *Mat****a*** *SMC3mcd1-L75K-LEU2:leu2-3,112 G418:MCD1-AID TIR1-CgTRP1*

*LacO-NAT::lys4 GFPLacI-HIS3:his3-11,15 ura3-52 bar1*

VG3939-7B *Mat****a*** *SMC3mcd1-L89K-LEU2:leu2-3,112 G418:MCD1-AID TIR1-CgTRP1*

*LacO-NAT::lys4 GFPLacI-HIS3:his3-11,15 ura3-52 bar1*

VG3952-14C *Mat****a*** *G418:SCC2-6MYC SMC3MCD1-URA3:ura3-52 SMC3-3V5-AID^608^ TIR1-CgTRP1 LacO-NAT::lys4 GFPLacI-HIS3:his3-11,15 leu2-3,112 bar1*

VG3953-17D *Mat****a*** *G418:SCC2-6MYC smc3-L1029R-MCD1-URA3:ura3-52 SMC3-3V5-AID^608^*

*TIR1-CgTRP1 LacO-NAT::lys4 GFPLacI-HIS3:his3-11,15 leu2-3,112 bar1*

VG3949-5C *Mat****a*** *G418:SCC3-6MYC SMC3-MCD1-URA3:ura3-52 SMC3-3V5-AID^608^*

*TIR1-CgTRP1 LacO-NAT::lys4 GFPLacI-HIS3:his3-11,15 leu2-3,112 bar1*

VG3950-8D *Mat****a*** *G418:SCC3-6MYC smc3-L1029R-MCD1-URA3:ura3-52 SMC3-3V5-AID^608^*

*TIR1-CgTRP1 LacO-NAT::lys4 GFPLacI-HIS3:his3-11,15 leu2-3,112 bar1*
