# Supplementary material for: Communication between distinct subunit interfaces of the cohesin complex promotes its topological entrapment of DNA
Source: eLife. 2019 Jun 4;8:e46347. doi: 10.7554/eLife.46347 (PMC6579514; doi:10.7554/eLife.46347)
Supplement: Supplementary file 2. [file elife-46347-supp2.docx]

**Supplementary file 2. Primers used for chromatin Immunoprecipitation (ChIP)**

***CEN*-distal *TRM1* *CAR***

VG641/VG642 (5’ AAA GAA GCA GGG GTA GAG AAG C 3’/ 5’ ATC AGC AGC GGT GAT TAC AC 3’)

VG625/VG626 (5’ CCA GCC AGA TAT TAT GGG CAA G 3’/ 5’ TCC TAG ACC TGG TGG AAA AAG C 3’)

VG627/VG628 (5’ CTT ATA GTT CCC AAG GCA TCC C 3’/ 5’ CCA AAC TCG TTG TTC TCG ATC C 3’)

VG629/VG630 (5’ TCT TCG TGC GCG AGG ATA TG 3’/ 5’ CGA ACA TTT CCG GAC AAT TGC 3’)

VG633/VG634 (5’ CCA ATC GTA TAA CGG AGC ATT GG 3’/ 5’ TGG TGC CAG AAG ATA TCA ACG 3’)

VG637/VG638 (5’ GCG CGA TAC CAT TCA GAA CAT C 3’/ 5’ TTA AAG TGG GCC CAA GAC CAG 3’)

VG639/VG640 (5’ GGG CAT CAC CTT TTC GTA AGC 3’ / 5’ TGA TCC ACC TGT CAT TTC GC 3’)

VG643/VG644 (5’ ACC CTT CTG TTC CAG TTT GC 3’/ 5’ GTT GCC TCC GGA GCA AAT TC 3’)

***Pericentric CARC1***

TE367/TE368 (5’ AAA GGT GCC CCA AGA AAA GG 3’/ 5’ AGC ACT TTA CTC GCT TGT GG 3’)

TE310/TE311 (5’ TAA AGC ATT GAC GCC AGA GC3’/ 5’ AAG TAC GCG TAC GAA GCA TC 3’)

TE306/TE307 (5’ CAA ACC ACC TCT TAC GTC GTT G3’/ 5’ TTT CGT GCA CTG CGT TCA AG 3’)

TE308/TE309 (5’ TCC TGG AAT GGA GAC CGT TTT C 3’/ 5’ AGC CGA CAA ATT TCG TGC AC 3’)

TE373/TE374 (5’ ACT TTG GTT TTC CGG TGT GC3’/ 5’ CCA GCG ATG AGA TGC GAA AAG 3’)

TE377/TE378 (5’ TCG CTT TTC GCA TCT CAT CG 3’/ 5’ AGC GGG CGG GTT ATA AAT AAC 3’)

TE533/TE534 (5’ ACC TTC TAC TTC CAT GCC GTT G 3’/ 5’ TGC GTG CCG ATG TAG AAT TG 3’)

***CEN* primers**

***CEN4* flanking primers**BR463/BR464 (5’ CAT GAT TCG CCG GGT AAA TA 3'/ 5’ GCA CTA GCC AAT TTA GCA CTT C 3')

BR465/BR466 (5' AAA ATG CCG AGG CTT TCA TA 3'/ 5' TGA CGA TAA AAC CGG AAG GA 3'

***CEN14* flanking primers**TE442/TE443 (5' TTA AAG CGG CTG AGT ATG GC 3'/ 5' TTT CCT CCA TTG CTC TCT ACG G 3')

TE446/TE447 (5' ACT AAA AGT GCC CCA AAC GG 3'/ 5' AGG AGC AGG GTA GCA TAA ACC 3')
